# The duration of embryo culture after mouse IVF differentially affects cardiovascular and metabolic health in male offspring

**DOI:** 10.1093/humrep/deaa205

**Published:** 2020-10-06

**Authors:** Anan Aljahdali, R K Raja Ili Airina, Miguel A Velazquez, Bhavwanti Sheth, Katrina Wallen, Clive Osmond, Adam J Watkins, Judith J Eckert, Neil R Smyth, Tom P Fleming

**Affiliations:** School of Biological Sciences, University of Southampton, Southampton General Hospital, Southampton SO16 6YD, UK; University of Jeddah, Jeddah, Saudi Arabia; School of Biological Sciences, University of Southampton, Southampton General Hospital, Southampton SO16 6YD, UK; School of Natural and Environmental Sciences, Newcastle University, Newcastle Upon Tyne NE1 7RU, UK; School of Biological Sciences, University of Southampton, Southampton General Hospital, Southampton SO16 6YD, UK; School of Biological Sciences, University of Southampton, Southampton General Hospital, Southampton SO16 6YD, UK; MRC Lifecourse Epidemiology Unit, University of Southampton, Southampton SO16 6YD, UK; Division of Child Health, Obstetrics and Gynaecology, Faculty of Medicine, University of Nottingham, Nottingham NG7 2UH, UK; Human Development and Health, Faculty of Medicine, University of Southampton, Southampton General Hospital, Southampton SO16 6YD, UK; School of Biological Sciences, University of Southampton, Southampton General Hospital, Southampton SO16 6YD, UK; School of Biological Sciences, University of Southampton, Southampton General Hospital, Southampton SO16 6YD, UK

**Keywords:** ART, mouse IVF and embryo culture, embryo transfer, blastocyst, DOHaD, offspring long-term health, growth trajectory, CV health, metabolic health, liver phenotype

## Abstract

**STUDY QUESTION:**

Do the long-term health outcomes following IVF differ depending upon the duration of embryo culture before transfer?

**SUMMARY ANSWER:**

Using a mouse model, we demonstrate that in male but not female offspring, adverse cardiovascular (CV) health was more likely with prolonged culture to the blastocyst stage, but metabolic dysfunction was more likely if embryo transfer (ET) occurred at the early cleavage stage.

**WHAT IS KNOWN ALREADY:**

ART associate with increased risk of adverse CV and metabolic health in offspring, and these findings have been confirmed in animal models in the absence of parental infertility issues. It is unclear which specific ART treatments may cause these risks. There is increasing use of blastocyst, versus cleavage-stage, transfer in clinical ART which does not appear to impair perinatal health of children born, but the longer-term health implications are unknown.

**STUDY DESIGN, SIZE, DURATION:**

Five mouse groups were generated comprising: (i) natural mating (NM)—naturally mated, non-superovulated and undisturbed gestation; (ii) IV-ET-2Cell—*in-vivo* derived two-cell embryos collected from superovulated mothers, with immediate ET to recipients; (iii) IVF-ET-2Cell—IVF generated embryos, from oocytes from superovulated mothers, cultured to the two-cell stage before ET to recipients; (iv) IV-ET-BL—*in-vivo* derived blastocysts collected from superovulated mothers, with immediate ET to recipients; (v) IVF-ET-BL—IVF generated embryos, from oocytes from superovulated mothers, cultured to the blastocyst stage before ET to recipients. Both male and female offspring were analysed for growth, CV and metabolic markers of health. There were 8–13 litters generated for each group for analyses; postnatal data were analysed by multilevel random effects regression to take account of between-mother and within-mother variation and litter size.

**PARTICIPANTS/MATERIALS, SETTINGS, METHODS:**

C57/BL6 female mice (3–4 weeks old) were used for oocyte production; CBA males for sperm with human tubal fluid medium were used for IVF. Embryos were transferred (ET) to MF1 pseudo-pregnant recipients at the two-cell stage or cultured in synthetic oviductal medium enriched with potassium medium to the blastocyst stage before ET. Control *in-vivo* embryos from C57BL6 × CBA matings were collected and immediately transferred at the two-cell or blastocyst stage. Postnatal assays included growth rate up to 27 weeks; systolic blood pressure (SBP) at 9, 15 and 21 weeks; lung and serum angiotensin-converting enzyme (ACE) activity at time of cull (27 weeks); glucose tolerance test (GTT; 27 weeks); basal glucose and insulin levels (27 weeks); and lipid accumulation in liver cryosections using Oil Red O imaging (27 weeks).

**MAIN RESULTS AND THE ROLE OF CHANCE:**

Blastocysts formed by IVF developed at a slower rate and comprised fewer cells that *in-vivo* generated blastocysts without culture (*P* < 0.05). Postnatal growth rate was increased in all four experimental treatments compared with NM group (*P* < 0.05). SBP, serum and lung ACE and heart/body weight were higher in IVF-ET-BL versus IVF-ET-2Cell males (*P* < 0.05) and higher than in other treatment groups, with SBP and lung ACE positively correlated (*P* < 0.05). Glucose handling (GTT AUC) was poorer and basal insulin levels were higher in IVF-ET-2Cell males than in IVF-ET-BL (*P* < 0.05) with the glucose:insulin ratio more negatively correlated with body weight in IVF-ET-2Cell males than in other groups. Liver/body weight and liver lipid droplet diameter and density in IVF-ET-2Cell males were higher than in IVF-ET-BL males (*P* < 0.05). IVF groups had poorer health characteristics than their *in-vivo* control groups, indicating that outcomes were not caused specifically by background techniques (superovulation, ET). No consistent health effects from duration of culture were identified in female offspring.

**LARGE SCALE DATA:**

N/A.

**LIMITATIONS, REASONS FOR CAUTION:**

Results from experimental animal models cannot be extrapolated to humans. Nevertheless, they are valuable to develop conceptual models, in this case, in the absence of confounding parental infertility, in assessing the safety of ART manipulations.

**WIDER IMPLICATIONS OF THE FINDINGS:**

The study indicates that longer duration of embryo culture after IVF up to blastocyst before ET leads to increased dysfunction of CV health in males compared with IVF and shorter cleavage-stage ET. However, the metabolic health of male offspring was poorer after shorter versus longer culture duration. This distinction indicates that the origin of CV and metabolic health phenotypes after ART may be different. The poorer metabolic health of males after cleavage-stage ET coincides with embryonic genome activation occurring at the time of ET.

**STUDY FUNDING/COMPETING INTEREST(S):**

This work was supported through the European Union FP7-CP-FP Epihealth programme (278418) and FP7-PEOPLE-2012-ITN EpiHealthNet programme (317146) to T.P.F., the Biotechnology and Biological Sciences Research Council (BBSRC) (BB/F007450/1) to T.P.F., and the Saudi government, University of Jeddah and King Abdulaziz University to A.A. The authors have no conflicts of interest to declare.

## Introduction

Infertility is thought to affect an estimated 186 million people globally (Inhorn and Patrizio, 2015). The development of ART has provided a partial clinical resolution to infertility with over 8 million children born to date, representing some 2–6% births in developed countries (Berntsen *et al.*, 2019; Crawford and Ledger, 2019). Although most IVF children appear healthy according to numerous systematic reviews, ART has been linked with a small increased risk of adverse obstetric and perinatal outcomes and birth defects compared with naturally conceived children (Pinborg *et al.*, 2013; Qin *et al.*, 2017; Berntsen *et al.*, 2019). In addition, longer-term health concerns of ART offspring have been associated mainly with: altered birthweight and growth (Ceelen *et al.*, 2009; Kleijkers *et al.*, 2014, 2016); increased risk of cardiovascular (CV) dysfunction comprising CV remodelling during pregnancy with vascular impairment and raised blood pressure evident in children through to at least adolescence (Ceelen *et al.*, 2008, 2009; Sakka *et al.*, 2010; Scherrer *et al.*, 2012; Valenzuela-Alcaraz *et al.*, 2013; Zhou *et al.*, 2014; von Arx *et al.*, 2015; Guo *et al.*, 2017; Meister *et al.*, 2018); and susceptibility to metabolic dysfunction including poorer glucose handling, insulin resistance and increased triglycerides (Sakka *et al.*, 2010; Chen *et al.*, 2014; Gkourogianni *et al.*, 2014; Pontesilli *et al.*, 2015; Guo *et al.*, 2017). In a minority of studies, impairment to neurological and cognitive health have also been reported (Sandin *et al.*, 2013; Liu *et al.*, 2017; Goldsmith *et al.*, 2018).

These sustained health effects have been linked to the ‘Developmental Origins of Health and Disease’ (DOHaD) concept suggesting environmental factors during development, especially the peri-conceptional period, may alter subsequent growth and morphogenesis through epigenetic, cellular and physiological processes (Feuer and Rinaudo, 2016; Fleming *et al.*, 2018). However, evaluation of ART children’s health is complex and confounded by the actual technologies and precise protocols applied in clinics, the gradual refinement in practice over time, and appropriateness of controls and comparator groups to distinguish between consequences mediated through parental infertility and ART practice (Berntsen *et al.*, 2019).

With these considerations in mind, animal models have been invaluable to assess effects of ART-associated technologies on long-term offspring health, removing confounders such as parental infertility and treatment variability and including suitable controls. These indicate ART treatments do indeed affect long-term health. Thus, IVF and/or mouse embryo culture and transfer result in offspring with altered growth trajectory, CV abnormalities and glucose/insulin dysfunction (Watkins *et al.*, 2007; Scott *et al.*, 2010; Le *et al.*, 2013; Rexhaj *et al.*, 2013; Chen *et al.*, 2014; Donjacour *et al.*, 2014; Feuer *et al.*, 2014; Ramirez-Perez *et al.*, 2014; Schenewerk *et al.*, 2014; Cerny *et al.*, 2017; Wang *et al.*, 2018).

In the last decade, there has been a gradual switch in ART practice from cleavage-stage embryo transfer (ET) to blastocyst stage ET to facilitate embryo selection and improve synchronicity with the uterine environment, despite the potential risk of increased embryo environmental perturbation. Whilst fresh blastocyst ET may marginally improve the live birth rate (Glujovsky *et al.*, 2016) without significantly affecting birthweight (De Vos *et al.*, 2018) or the risk of adverse perinatal outcomes (Shi *et al.*, 2019), it is unknown whether extended culture negatively impacts on later health status. In the current study, we have used a mouse model to assess the effect of cleavage or blastocyst ET on offspring health across a range of growth, CV and metabolic criteria.

## Materials and methods

### Animals

Animal treatments were conducted in accordance with the UK Home Office Animal (Scientific procedure) Act 1986 and local ethics committee at the University of Southampton. CBA male and C57/BL6 female mice (source of embryos) and MF1 females (pseudo-pregnant recipients) were bred in-house (University of Southampton, Biomedical Research Facility) on a 07:00–19:00 light cycle, 24°C and fed *ad libitum* from weaning on a standard chow diet (Special Diet Service, Ltd, Witham, Essex, UK) and water.

### Embryo production and treatment

Virgin female C57/BL6 mice (3–4 weeks old) were superovulated by i.p. injection of 5 IU pregnant mare’s serum gonadotropin (PMSG, Intervet, Cambridge, UK) and 46 h later, 5 IU hCG (Intervet, Cambridge, UK). For *in-vivo* produced embryos, females were housed overnight with CBA males. Plug positive females at embryonic day 0.5 (E0.5) (i.e. midday of plug detection day) were housed individually and, at E1.5 and E3.5, females were killed by cervical dislocation and two-cell embryos and blastocysts were flushed from dissected oviducts and uteri, respectively, into pre-warmed H6 medium supplemented with 4 mg/ml bovine serum albumin (BSA, A3311, Sigma, UK) (Nasr-Esfahani *et al.*, 1990). Some females were also naturally mated without superovulation.

For IVF embryo production, sperm was retrieved from the cauda epididymis of CBA males (8 weeks old) and placed into 90 µl sperm pre-incubation medium TYH-MBCD (Takeo and Nakagata, 2011) made in-house and equilibrated for 1 h at 37°C in 5% CO_2_ in air. C57/BL6 females were superovulated as above and cumulus masses, collected from the oviduct ampulla 13 h post-hCG injection, were placed directly into 200 µl fertilisation drop containing human tubal fluid (HTF) medium made in-house with 1.0 mM reduced glutathione (GSH, Sigma: G4251). Sperm (3-5 µl from pre-equilibrated TYH-MBCD drop) were added to the fertilisation drop and incubated for 3–4 h to allow fertilisation to occur (Ishizuka *et al.*, 2013). Presumptive zygotes were washed through four drops HTF medium without GSH and then cultured in the fourth drop under oil at 37°C and 5% CO_2_ in air to the next day (E1.5) before calculating the fertilisation rate. IVF embryos (two-cell stage) were then divided into two groups, the first was washed in pre-warmed M2 medium (Sigma; Cat No. M7167) before transfer to E0.5 MF1 pseudo-pregnant mothers. The second group was cultured in potassium simplex optimised medium with amino acids and BSA (synthetic oviductal medium enriched with potassium; Sigma-Aldrich) (Biggers *et al.*, 2005) at 37°C in 5% CO_2_ in air to the blastocyst stage before washing in M2 medium and transfer to E2.5 MF1 pseudo-pregnant mothers.

*In-vivo* and IVF-generated blastocyst trophectoderm (TE) and inner cell mass (ICM) cell numbers were determined by differential nuclear staining as described (Handyside and Hunter, 1984) with modifications (Velazquez *et al.*, 2018).

### Embryo transfer

ET was performed by flank laparotomy in pseudo-pregnant MF1 recipients (7–8.5 weeks) obtained by mating with vasectomised MF1 males. Two-cell embryos and blastocysts were washed three times in M2 medium prior to ET into the oviduct and uteri, respectively, in minimal medium, as previously described (Velazquez *et al.*, 2018). Recipients were anaesthetised by a single intraperitoneal injection of Ketamine (50 mg/kg, Ketaset, Pfizer, UK) and Xylazine (10 mg/kg, Rompun, Bayer, UK). Embryos were transferred (19.7 ± 6.05 per recipient) in equal numbers into both maternal tracts with separate recipients used for different treatments, as below. After transfer, exposed tracts were placed back into the abdominal cavity, the peritoneum was sutured, and the skin was closed with wound clips. Recipients were then kept individually in a clean cage in a warm room (28–30°C) to recover from anaesthesia. Females were then housed in a quiet room for the rest of their pregnancy and lactation. Litter size was adjusted to up to 8 per dam at birth with similar numbers of males and females.

### Animal treatment groups

**Figure 1.**
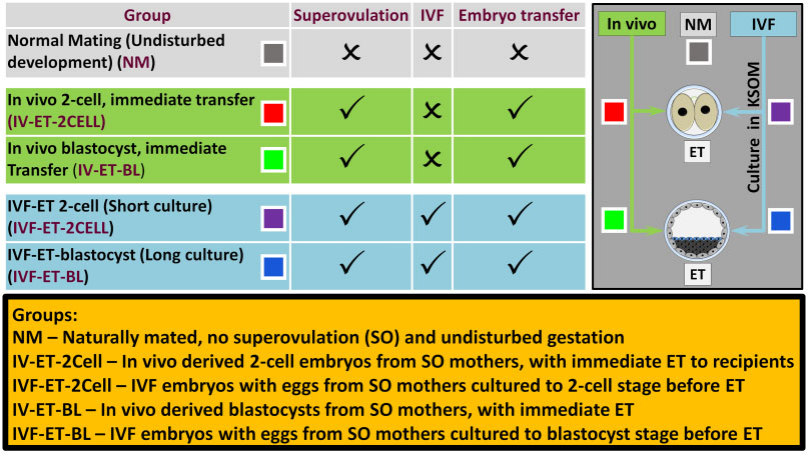
**Experimental design showing the five treatment groups used**.

Eight to 13 litters were generated from each of five treatments with groups termed as follows: (i) natural mating (NM)—naturally mated, non-superovulated and undisturbed gestation; (ii) IV-ET-2Cell—*in-vivo* derived two-cell embryos collected from superovulated mothers, with immediate ET to recipients; (iii) IVF-ET-2Cell—IVF generated embryos with oocytes from superovulated mothers cultured to the two-cell stage before ET to recipients; (iv) IV-ET-BL—*in vivo* derived blastocysts collected from superovulated mothers, with immediate ET to recipients; (v) IVF-ET-BL—IVF generated embryos with oocytes from superovulated mothers cultured to the blastocyst stage before ET to recipients. These treatment groups are shown in Fig. 1.

### Offspring analysis

All offspring from the five treatment groups were weaned at 3 weeks and males and females were caged separately per litter. Offspring body weight was recorded weekly for 27 weeks. Systolic blood pressure (SBP) was measured at postnatal weeks 9, 15 and 21 by tail-cuff plethysmography with Non-Invasive Blood Pressure Monitor (NIBP-8, Columbus Instruments, Columbus, OH, USA) in a pre-warmed room (28–30°C) to which mice were acclimatised for 90 min, as described previously (Velazquez *et al.*, 2018). Five SBP readings with good waveforms and good overall quality were taken per mouse, and the mean value of the three middle readings was calculated and recorded. Heart rate was monitored as an indicator of stress, and if reaching >500 beats per minute, SPB readings were delayed until heart rate reduced. Glucose tolerance tests (GTT) were conducted at postnatal week 27 in unrestrained conscious mice after 15 h overnight fast, with access to water. A standard protocol for GTT using a blood glucose meter (Accu-Chek Aviva, Roche Diagnostics GmbH, Germany) to measure blood glucose in small drops collected by tail tipping was employed. Topical anaesthetic cream (Lidocaine 5%, Teva, UK) was applied to the tail 20 min before starting the GTT. After recording of fasting glucose level (0 min), a glucose (G8270, Sigma) solution (20%, in sterile distilled water) was i.p. injected at a dose of 2 g/kg. Blood glucose levels were measured at 15, 30, 60 and 120 min after glucose administration. Area under the curve (AUC) values were calculated by the trapezoidal rule (Matthews *et al.*, 1990). Organ Allometry was determined 2 days after GTT: mice were sacrificed by cervical dislocation, blood was collected by heart puncture and organs (i.e. liver, heart, left and right kidneys, lung and spleen) were weighed, snap frozen in liquid nitrogen and stored at −80°C. Blood samples were centrifuged at 4°C for serum collection and stored at −80°C.

### Angiotensin-converting enzyme activity

The method was used as previously (Watkins *et al.*, 2006, 2007) to measure serum and lung angiotensin-converting enzyme (ACE) activities, the classical enzyme regulator of the renin-angiotensin system converting Angiotensin I to the vasopressor Angiotensin II (Li *et al.*, 2017). The assay is based on the colourimetric determination of hippurate with cyanuric chloride/dioxan reagent. Briefly, for serum ACE activity, samples were incubated in hippuryl-l-histidyl-l-leucine (HHL; Sigma) solution in H_3_PO_3_ buffer at 37°C, the reaction was terminated with HCl (Sigma) followed by addition of cyanuric chloride (Sigma) in 1,4-dioxan (Sigma) for yellow colouration to develop. Four replicates per sample were analysed using a plate reader (Varioskan Flash Multimode Reader; Thermo Scientific) at 380 nm. Negative controls comprised addition of HCl before HHL. A Hippurate standard curve (20–100 µM) was prepared from 112 mg Hippuric acid (Sigma) solution in 250 ml 20 mmol/l NaOH, treated as samples except the addition of HHL. Each of the four replicates per sample were analysed in duplicate, and the average of these eight readings taken. For lung ACE activity, lung samples of 50 ± 1 mg were homogenised in 300 μl ice-cold boric buffer (H_3_BO_3_, 2M NaCl, pH 8.3; Sigma) with a PowerGen homogeniser, centrifuged at 16 400 rpm for 10 min at 4°C and the supernatant was removed and stored at −80°C. Pellets were homogenised in 300 μl buffer and centrifuged, and the supernatant was removed and stored. Duplicate analysis of four replicate supernatants per sample was analysed as described for serum ACE activity. Total protein content of samples was measured using a BioRad kit. Serum ACE activity was expressed as amount (in µM) of hippurate formed per millilitre of serum per minute; lung ACE activity was expressed as amount (in nanomolar) of hippurate formed per milligram of protein per minute. Serum and lung samples were selected at 27 weeks from the same offspring at the middle weight across litters from the five treatment groups (one male and one female from each of 7–9 mothers per treatment) and stored frozen. These same offspring were used for serum glucose and insulin assays and for the liver lipid metabolism assay.

### Serum glucose and insulin analysis

Glucose concentration in offspring serum was measured using the glucometer as described in the GTT procedure. Serum insulin concentration was determined using an ELISA kit (Mercodia, Sweden, Mouse: 10-1247-01) based on the manufacturer’s instructions. Briefly, 10 µl of each calibrator 0, 1, 2, 3, 4 and 5 and serum samples were incubated in coated microplate wells with 100 µl enzyme conjugate solution on a plate shaker at room temperature at 750 rpm for 2 h, before washing in 350 µl of wash buffer repeated five times, before addition of 200 µl TMB substrate and incubation for 15 min, before addition of 50 µl stop solution. Absorbance was measured at 450 nm using a Varioskan Flash Multimode Reader (Thermo Scientific). Standard deviation and coefficient of variance were calculated for each sample run in duplicate in three plates and mean insulin values were calculated. The glucose/insulin ratio (G:I) ratio was calculated to assess insulin resistance (McAuley *et al.*, 2001). A total of 6–8 samples from each treatment, both male and female and each from a separate mother, were used for combined glucose and insulin analyses.

### Liver morphometrics and metabolism

Frozen-stored adult offspring median lobe liver samples were embedded in OCT-compound and cryosections at 7 µm were generated and stained with Oil Red O to visualise lipid accumulation and Mayer’s Haematoxylin as counterstain before mounting in aqueous medium and applying coverslips. Images of sections were analysed and photographed using an Olympus dotSlide Virtual Microscopy System with an Olympus BX61 Microscope Frame at 10× magnification. Images (3 per liver sample) were quantified using Fiji software for red-stained lipid accumulation with the Watershed tool applied to separate grouped lipid droplets. A total of 6–9 offspring from each treatment, both male and female and each from a separate mother, were used for liver analyses. 

### Statistics

Statistical analyses were performed with the IBM SPSS Statistics software, version 21 (IBM Corporation) and significance was taken as *P* ≤ 0.05. If a *P*-value of between 0.1 and 0.05 was observed, a trend was assumed to exist. Blastocyst cell number, rates of blastocyst development and ET outcome (i.e. pregnancy rate, ET efficiency and litter size) were analysed using a one-way ANOVA followed by a pairwise *t*-test with Bonferroni correction analysis. Percentage data were arcsine transformed before ANOVA analysis. Postnatal data, comprising offspring weights, SBP, GTT, organ weights and ratios, post-culling serum glucose and insulin, serum and lung ACE activities and liver lipid accumulation data, were analysed using multilevel random effects regression models to compare treatment groups (Kwong *et al.*, 2004) and to analyse relationships between different readouts (i.e. correlations) within each treatment group (Velazquez *et al.*, 2018). All postnatal data were converted to Z-scores before being analysed with the regression models which took into account between-mother and within-mother variation and litter size (Kwong *et al.*, 2004; Watkins *et al.*, 2008).

## Results

### IVF and embryo culture delay blastocyst development and reduce cell proliferation

**Figure 2.**
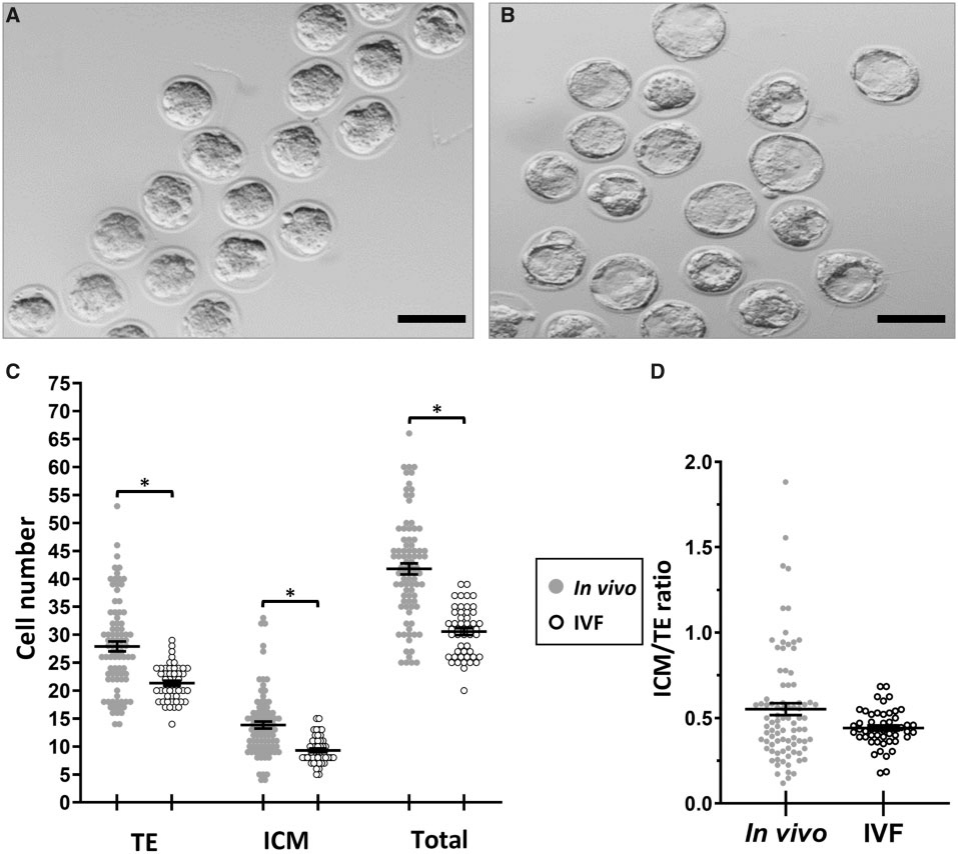
**Effect of IVF and prolonged embryo culture on blastocyst development cell number**. IVF embryos at E3.5 comprise morulae (**A**) and at E4.5 comprise blastocysts (**B**); bar = 100 µm. (**C**) IVF embryos (n = 50) have fewer cells than *in-vivo* embryos (n = 87) at the blastocyst stage. Mean (±SEM) blastocyst cell number for IVF compared with *in-vivo* embryos (*P* < 0.05). (**D**) Mean (±SEM) ICM/TE ratio of blastocysts. * *P* < 0.05.

Routine analysis of IVF embryo development was conducted throughout the study, with oocytes (n = 1720) collected from 40 superovulated dams, used in 14 separate IVF experiments, leading to an overall mean success rate of two-cell embryo formation of 92%, and from those allocated to culture, 81% formed morulae and 72% developed to blastocysts. The developmental rate of IVF embryos was compared with *in-vivo* embryos (superovulated; naturally mated; develop *in vivo*; collected at E3.5). IVF embryos developed more slowly and only reached the morula stage at E3.5 whilst *in-vivo* embryos had become expanding blastocysts by then (Table I). IVF embryos became expanding blastocysts by E4.5 (Fig. 2A and B; Table II). Some IVF and *in-vivo* mid-expanded blastocysts at E4.5 and E3.5 days, respectively, were subjected to differential cell staining which showed increased TE, ICM and total cell numbers in *in-vivo* versus IVF embryos (*P* < 0.05) although the ICM:TE ratio did not differ between the two groups (Fig. 2C and D). IVF and prolonged culture therefore delayed blastocyst formation and reduced associated proliferation of both cell lineages compared with *in-vivo* development.

**Table I:** Developmental rate of *in vivo* and IVF embryos at E3.5 and E4.5.

Group	Number (%)						
	Mean (SD ± SEM) from each dam						
	Dam number	Embryo number	Morula	Early blastocyst	Mid blastocyst	Late blastocyst	Arrested (early cleavage)
in vivo	22	469	15 (3.2)	40 (8.5)	114 (24.3)	295 (62.9)	5 (1.1)
E3.5			0.71 (0.72 ± 0.15)	1.9 (0.89 ± 0.19)	5.43 (1.29 ± 0.27)	14.05 (3.31 ± 0.7)	0.24 (0.44 ± 0.09)

IVF	40	1076^1^	876 (81.4)	0	0	0	200 (18.6)
E3.5			62.57 (38.14 ± 6.03)				14.28 (5.69 ± 0.9)
IVF			109 (12.4)^2^	51 (5.8)^2^	83 (9.5)^2^	633 (72.3)^2^	0
E4.5			7.78 (5.1 ± 0.81)	3.64 (1.98 ± 0.31)	5.92 (3.19 ± 0.5)	45.21 (28.12 ± 4.45)	

Early blastocyst has a blastocoel volume less than half of the total embryo volume.

Mid blastocyst has a blastocoel volume equal to or larger than the total embryo volume.

Late blastocyst blastocoel fully expanded within the embryo whilst the zona pellucida (ZP) is thinning.

1 Number of 2-cell embryos cultured after IVF.

2 Per cent of morulae at E3.5.

**Table II:** **Offspring production criteria for the five treatment groups as shown in**  **Fig. 1**.

Treatment Group	**ET pregnancy rate** ^1^ **% (dam numbers)**	**ET efficiency** ^2^ **% (pups/ embryos transferred)**	**Birth litter size** ^3^ **Mean (SD ± SEM) [litter number]**	Offspring number	No. male/ female pups	Ratio Male:Female
NM	N/A	N/A	8 (1.33 ± 0.42)^a^ [10]	80	40/40	1
IV-ET-2Cell	88.9 (8/9)	31.7 (57/180)^a^	7.12 (4.36 ± 1.54)^b^ [8]	57	32/25	1.3
IVF-ET-2Cell	73.3 (11/15)	16.7 (75/450)^a^	8.33 (3.74 ± 1.25)^a1^ [9]	75	42/33	1.2
IV-ET-BL	81.8 (9/11)^a^	30.5 (47/154)^a^	5.88 (1.73 ± 0.61)^b^ [8]	47	22/25	0.9
IVF-ET-BL	48.3 (14/29)^b^	9.1 (42/464)^b^	3.23 (1.79 ± 0.5)^b^^,^^b1^ [13]	42	26/16	1.6

Data were analysed using ANOVA (mean± SEM).

1 Dams that gave birth/total number of ETs performed.

2 Total number of pups at birth (before litter size correction)/total embryos transferred (7–15 per side).

3 Calculated on dams with live pups at birth (before litter size correction).

a,b; a1,b1 Within a column, values with different letters are significantly different (*P* < 0.05).

### Postnatal offspring from ART treatments display increased body weight

To study the effect of ART and embryo culture duration on postnatal development, we generated the five treatment groups as shown in Fig. 1 with the offspring production criteria shown in Table II. The ET pregnancy rate (% dams giving birth) was significantly higher in the IV-ET-BL group compared with IVF-ET-BL, otherwise no differences were found between groups (Table II). ET efficiency (pups generated per numbers of embryos transferred) was lower in IVF-ET-BL than other groups. Litter size in the ET groups IV-ET-2Cell, IV-ET-BL and IVF-ET-BL was lower than the NM group. The IVF-ET-BL litter size was also lower than the IVF-ET-2Cell group. Male:female ratio was not different between any of the treatment groups (Table II).

**Figure 3.**
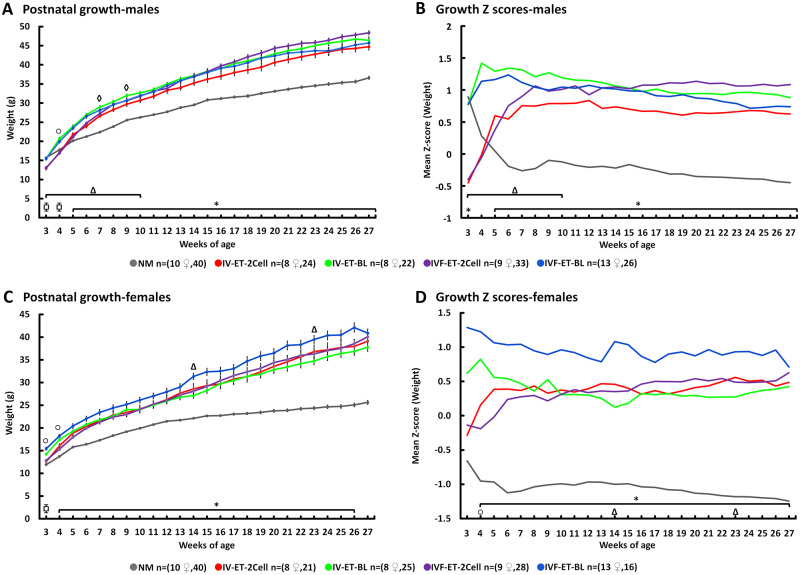
**Effect of ART techniques on growth of offspring.** Body weight and Z-score analysis in male (**A** and **B**) and female (**C** and **D**) offspring. Multilevel random effects regression analysis. *Indicates a significant difference (*P* < 0.05) between NM and other treatments; ⧮ denotes NM vs. (IV-ET-2Cell, IVF-ET-2Cell and IVF-ET-BL) at Week 3 and NM vs. IV-ET-BL at Week 4 (*P* ≤ 0.05), Δ indicates IV-ET-2-Cell vs. IV-ET-BL and ○ indicates IVF-ET-BL vs. IVF-ET-2Cell (*P* ≤ 0.05). Mean (±SEM) body weight from 3 to 27 weeks (from 8 to 13 litters); n of mothers or foster mothers ♀, n of offspring.

Male and female offspring body weight differences between groups were analysed from weaning through to Week 27, taking into consideration litter size and individual maternal origin. All four ET groups were significantly heavier compared with the NM control group, evident from Week 5 (males) and 4 (females) through to Week 27 (Fig. 3A and C). Z-score plots confirmed increased body weight for all ET groups compared with the NM group up to Week 27 (Fig. 3B and D). Generally, weight differences between different ET groups were minimal and are itemised in the Fig. 3 legend. Notably, IVF-ET-BL female mean weight was heavier than other ET groups throughout the 27-week period (Fig. 3C and D). Thus, the combined techniques of ART (superovulation, IVF, culture, transfer, recipient gestation) in our model, or just some of them (minimal superovulation, transfer, recipient gestation), resulted in sustained increase in postnatal weight in both sexes compared with natural, unstimulated reproduction.

### Male offspring from IVF and prolonged culture before ET develop CV dysfunction

**Figure 4.**
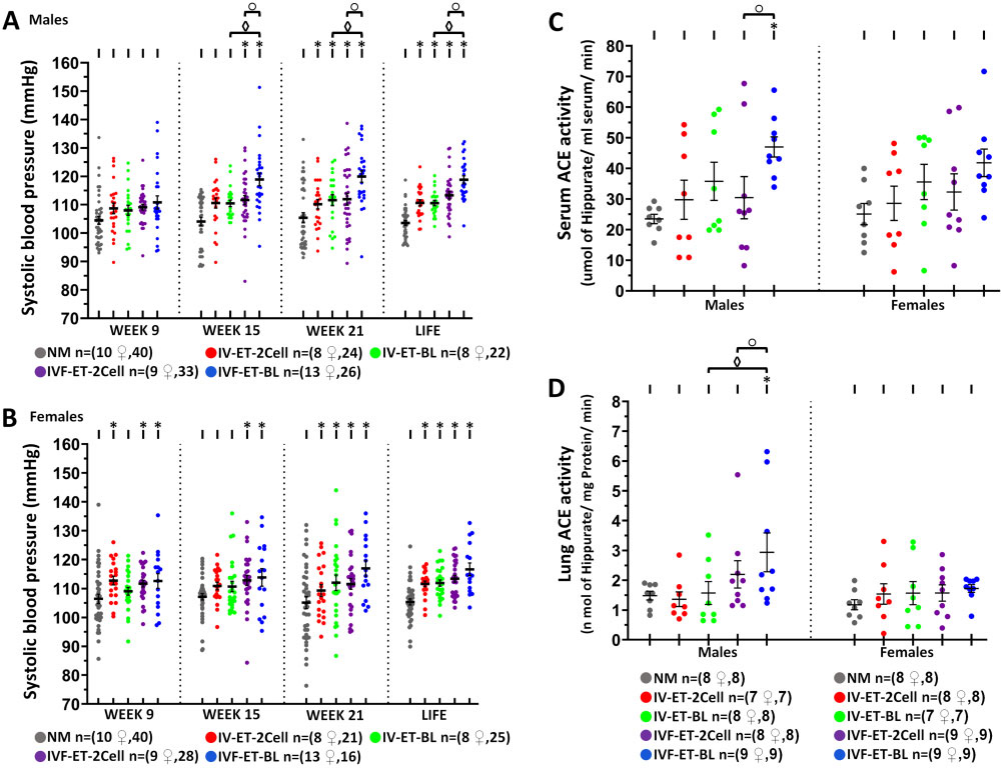
**Effect of ART techniques on cardiovascular function in offspring.** Postnatal systolic blood pressure (SBP) at indicated weeks of age and mean of these for individual offspring (LIFE), in male (**A**) and female (**B**) offspring; mean (±SEM) from 8 to 13 litters. Serum ACE activity (**C**) and lung ACE activity (**D**) in male and female offspring; mean (±SEM) from 7 to 9 litters per treatment. Multilevel random effects regression analysis. *Indicates a significant difference (*P* < 0.05) between NM and selected groups; ◊ indicates IV-ET-BL vs. IVF-ET-BL, and ○ indicates IVF-ET-BL vs. IVF-ET-2Cell differences (*P* < 0.05). n of mothers or foster mothers ♀, n of offspring. ACE, angiotensin-converting enzyme.

SBP was determined at 9, 15 and 21 weeks and the mean of these also recorded as LIFE (Fig. 4). In males, mean SBP for all time points was consistently highest in IVF-ET-BL, reduced in IVF-ET-2Cell, the two IV-ET control groups and lowest in the NM group (Fig. 4A). IVF-ET-BL male SBP was increased at Weeks 15, 21 and LIFE compared with IVF-ET-2Cell (*P* = 0.032, 0.034 and 0.017, respectively) and with IV-ET-BL (*P* = 0.003, 0.014 and 0.001, respectively) (Fig. 4A). In females, although a similar SBP pattern existed across treatment groups, the differences were not significant between ET groups (Fig. 4B). However, NM females showed significant lower SBP than females in IV-ET and IVF-ET groups at Weeks 15, 21 and LIFE (*P* < 0.05).

Serum and lung ACE activity, known to associate with increased SBP (Li *et al.*, 2017), were further measured in offspring. Male IVF-ET-BL offspring recorded the highest serum and lung ACE activity, both being higher (*P* < 0.05) than in the IVF-ET-2Cell males (Fig. 4C and D). IVF-ET-BL lung ACE in males was also higher than the control IV-ET-BL males (*P* < 0.05) (Fig. 4D). However, ACE activities were not different across groups in female offspring (Fig. 4C and D). Correlation analysis of SBP and ACE activity revealed a significant positive correlation between both SBP 21 weeks and SBP LIFE with Lung ACE activity in male IVF-ET-BL offspring but not in females nor in any other treatment group (Table III).

**Table III:** Phenotypic correlations between different offspring outcomes across treatments.

	Natural mating		IV-ET-2cell		IV-ET-BL		IVF-ET-2cell		IVF-ET-BL	
	Male (n = 8)	Female (n = 8)	Male (n = 6–8)	Female (n = 7–8)	Male (n = 6–8)	Female (n = 6–7)	Male (n = 8–9)	Female (n = 7–9)	Male (n = 7–9)	Female (n = 7–9)
Cardiovascular phenotype										
SBP wk21 − Lung ACE	−0.190	0.415	0.556	0.370	0.184	0.130	−0.026	0.223	0.902*	−0.119
SBP LIFE − Lung ACE	0.118	0.107	0.415	0.353	0.693^$^	0.328	0.101	0.195	0.714*	−0.177
G:I and body weight										
G:I ratio − BW3	−0.305	−0.351	−0.211	−0.566	−0.403	0.080	−0.224	0.160	−0.676^$^	−0.268
G:I ratio − BW9	0.111	0.169	−0.621	−0.649	0.936*	0.789^$^	−0.806*	−0.073	−0.664	−0.013
G:I ratio − BW15	0.128	−0.303	−0.761^$^	−0.735^$^	−0.904*	0.749^$^	−0.807*	−0.309	−0.700^$^	−0.053
G:I ratio − BW21	0.287	−0.184	−0.848*	−0.710^$^	−0.871*	0.664	−0.812*	0.008	−0.748^$^	0.122
G:I ratio − BW27	0.001	−0.448	−0.832*	−0.883*	−0.768^$^	0.910*	−0.935*	−0.026	−0.806*	−0.741^$^
G:I ratio − FG	−0.523	−0.183	−0.040	−0.431	−0.651	0.471	−0.640^$^	−0.090	0.437	0.198
G:I ratio − GTT 15 min	−0.010	−0.021	−0.082	−0.282	−0.383	0.679	−0.806*	0.137	−0.767*	0.234
G:I ratio − GTT 120 min	0.298	−0.350	−0.753^$^	−0.547	−0.432	0.927*	−0.648^$^	0.043	−0.707^$^	−0.201
Insulin – AUC	0.023	−0.111	−0.013	0.208	0.252	−0.912*	0.860*	−0.315	0.659	0.420
G:I ratio − AUC	−0.195	−0.107	−0.263	−0.371	−0.540	0.960*	−0.862*	0.116	−0.819*	−0.530

SBP LIFE, average (SBP9, SBP15 and SBP21); ACE, angiotensin-converting enzyme; G:I G:I ratio, Serum glucose: Serum insulin ratio; BW, body weight as specific week; AUC, area under the curve for GTT test; Insulin, Serum insulin; FG, fasting glucose.

$ *P* < 0.1;

* *P* < 0.05.

The combined techniques of ART (superovulation, IVF, culture, ET, recipient gestation) therefore contribute to adverse postnatal CV health compared with natural unstimulated reproduction but with prolonged versus short embryo culture exacerbating these effects in male offspring.

### Male offspring from IVF and short culture before ET develop impaired glucose and insulin metabolism

**Figure 5.**
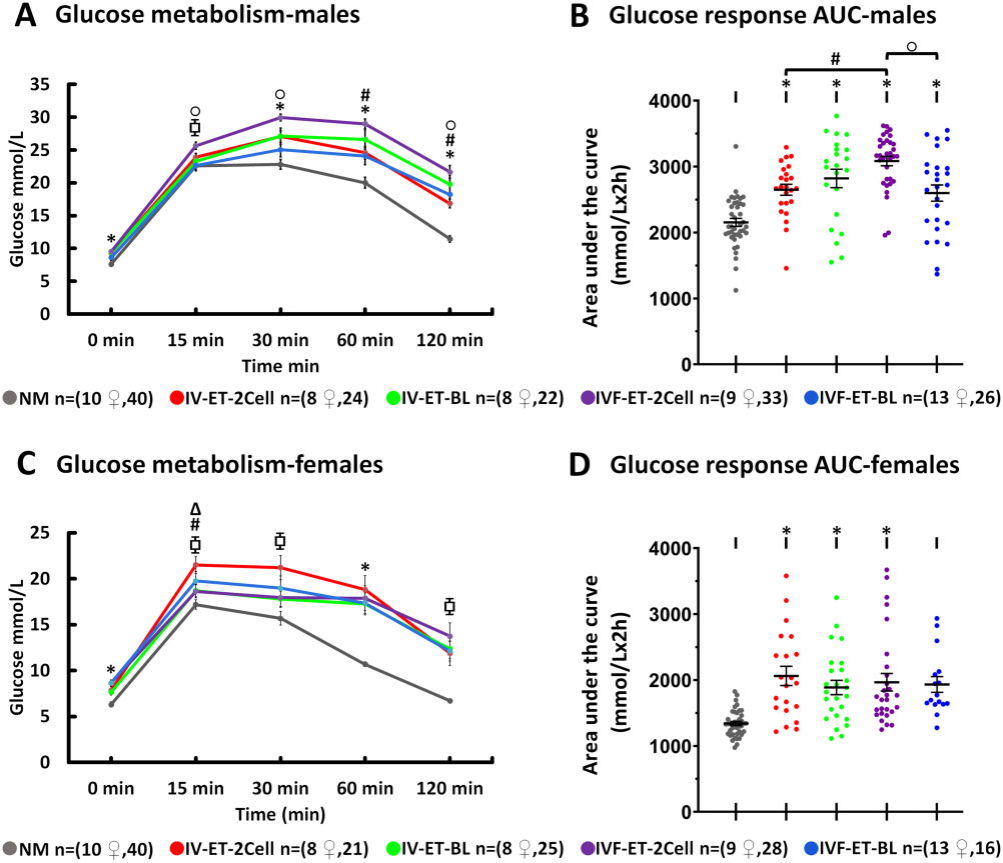
**Effect of ART techniques on glucose metabolism in offspring.** Intraperitoneal GTT at 0, 15, 30, 60 and 120 min and AUC in male (**A** and **B**) and female (**C** and **D**) offspring; mean (±SEM) in 8–13 litters per treatment. Multilevel random effects regression analysis. *Indicates a significant difference (*P* < 0.05) between NM and selected groups; Δ indicates IV-ET-2-Cell vs. IV-ET-BL, # indicates IV-ET-2Cell vs. IVF-ET-2Cell, and ○ indicates IVF-ET-BL vs. IVF-ET-2Cell differences (*P* ≤ 0.05). n of mothers or foster mothers ♀, n of offspring.

Glucose metabolism of offspring was assessed by glucose tolerance test (GTT) at postnatal Week 27. Male offspring fasting glucose level (i.e. 0 min) and after 15, 30 min, 1 and 2 hr of i.p glucose injection showed all treatment groups to have significantly slower recoveries and larger AUC than the NM control group (Fig. 5A and B). Glucose recovery and AUC for IVF-ET-2Cell was poorer compared with both IV-ET-2Cell (*P* = 0.05–0.004) and IVF-ET-BL males (*P* = 0.03–0.003). In female offspring, fasting glucose level, glucose recovery and AUC also appeared to be poorer in treatment groups compared with the NM control although the differences were not always significant. No significant differences were detected between the four treatment groups in females (Fig. 5C and D).

**Figure 6.**
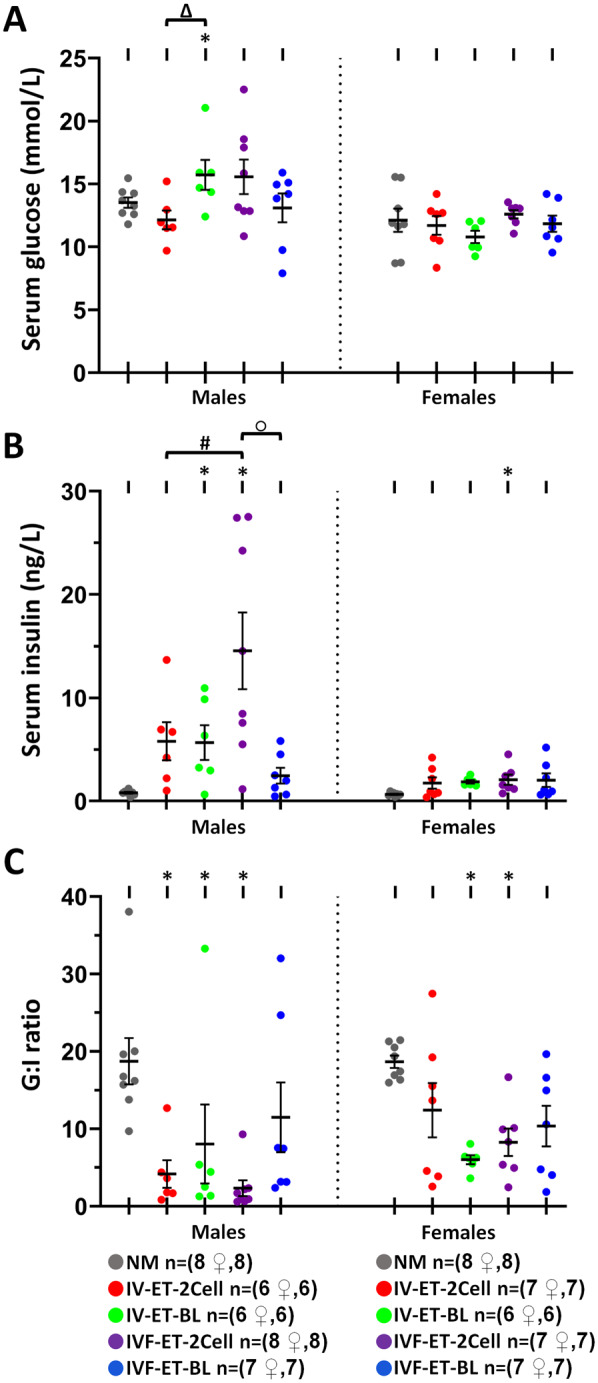
**Effect of ART techniques on glucose and insulin levels in offspring.** Serum glucose (**A**), serum insulin (**B**) and G:I ratio (**C**) in male and female offspring; mean (±SEM) from 6 to 8 litters per treatment. Multilevel random effects regression analysis. *Indicates a significant difference (*P* < 0.05) between NM and selected groups; Δ indicates IV-ET-2-Cell vs. IV-ET-BL, # indicates IV-ET-2Cell vs. IVF-ET-2Cell, and ○ indicates IVF-ET-BL vs. IVF-ET-2Cell differences (*P* ≤ 0.05). n of mothers or foster mothers ♀, n of offspring.

Serum samples collected at 27 weeks during animal culling were used to measure insulin and glucose levels and the glucose:insulin ratio (G:I), a measure of insulin effectiveness in glucose homeostasis. In male offspring, glucose levels were similar across treatments with IV-ET-BL higher than IV-ET-2Cell and NM (*P* < 0.05; Fig. 6A). In contrast, insulin levels differed substantially across treatments with IVF-ET-2Cell males being significantly higher than all other groups (*P* < 0.05; Fig. 6B). The lowest insulin level was in NM males which led to the highest G:I ratio in NM males and this was significantly higher than in IV-ET-2Cell, IVF-ET-2Cell and IV-ET-BL groups (*P* = 0.005, *P* = 0.001 and *P* = 0.038, respectively; Fig. 6C). Female serum glucose was unchanged across treatments (Fig. 6A) while insulin was lowest in the NM group and significantly raised in IVF-ET-2Cell females (*P* < 0.05; Fig. 6B), resulting in G:I ratio highest in NM females, as in males, and significantly higher than in IV-ET-BL and IVF-ET-2Cell females (*P* < 0.05; Fig. 6C).

Metabolic outcomes were analysed for possible associations with other phenotypes; the G:I ratio in particular was found to be significantly negatively correlated both with body weight throughout postnatal life and with AUC from the GTT in the IVF-ET-2Cell male but not female offspring (Table III). Other groups with ET treatment also showed weaker associations between these parameters but there were no associations on the NM group (Table III).

The combined techniques of ART (superovulation, IVF, culture, ET, recipient gestation) therefore contribute to adverse postnatal metabolic health as measured by glucose homeostasis compared with natural unstimulated reproduction. Here, evidence of insulin resistance was most pronounced after short embryo culture particularly in male offspring.

### Offspring from IVF and short culture before ET develop increased lipid accumulation in liver

**Figure 7.**
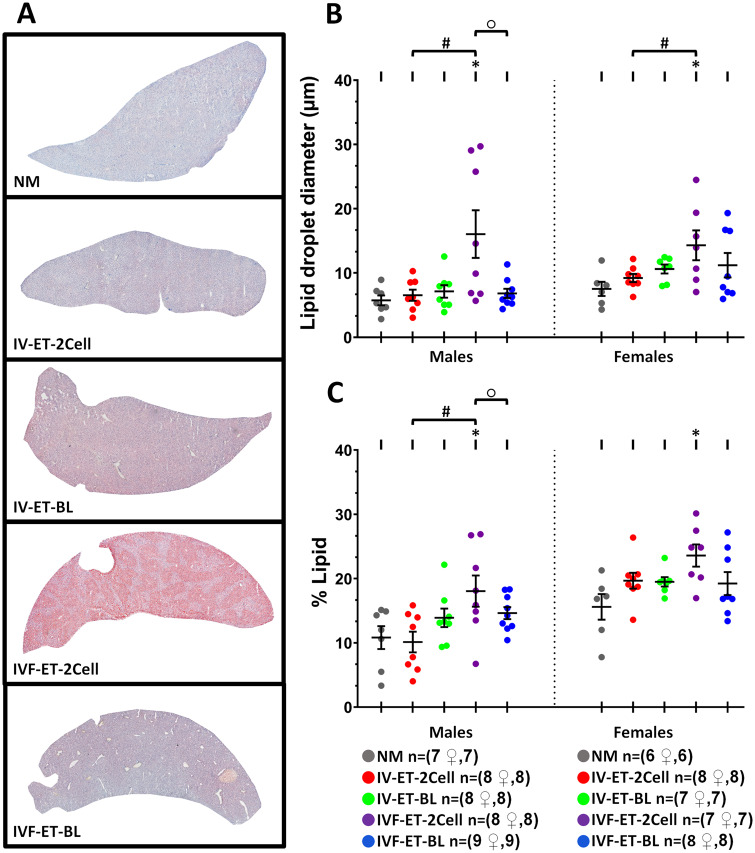
**Effect of ART techniques on lipid accumulation in offspring liver.** Representative images of liver cryosections from male offspring stained with Oil Red O from each treatment group (**A**). Average lipid droplet diameter (**B**) and percentage lipid-stained area (**C**) in male and female offspring; mean (±SEM) from 6 to 9 litters per treatment. *Indicates a significant difference (*P* < 0.05) between NM and selected groups; ○ indicates IVF-ET-BL vs. IVF-ET-2Cell (**B**, *P* = 0.015; **C**, *t* = 0.065), and # indicates IV-ET-2Cell vs. IVF-ET-2Cell differences (*P* < 0.05). n of mothers or foster mothers ♀, n of offspring.

Metabolic health of offspring was also assessed by analysis of lipid accumulation in liver cryosections stained with Oil Red O using organs stored at 27 weeks at culling. Representative images of lipid accumulation in male liver sections are shown in Fig. 7A. Lipid droplet size was increased in IVF-ET-2Cell offspring relative to other groups and especially in males. IVF-ET-2Cell lipid size was increased compared with IVF-ET-BL (*P* = 0.015) and with IV-ET-2Cell (*P* = 0.015) in males (Fig. 7B). Moreover, the relative percentage area of lipid accumulation was increased in IVF-ET-2Cell versus IVF-ET-BL at trend level (*t* = 0.065) and versus control IV-ET-2Cell (*P* = 0.003) in males (Fig. 7C). Thus, IVF and transfer after short rather than long culture contribute to adverse liver lipid accumulation as well as impaired glucose-insulin metabolism, especially in males.

### Postnatal offspring from ART treatments display altered organ allometry

**Figure 8.**
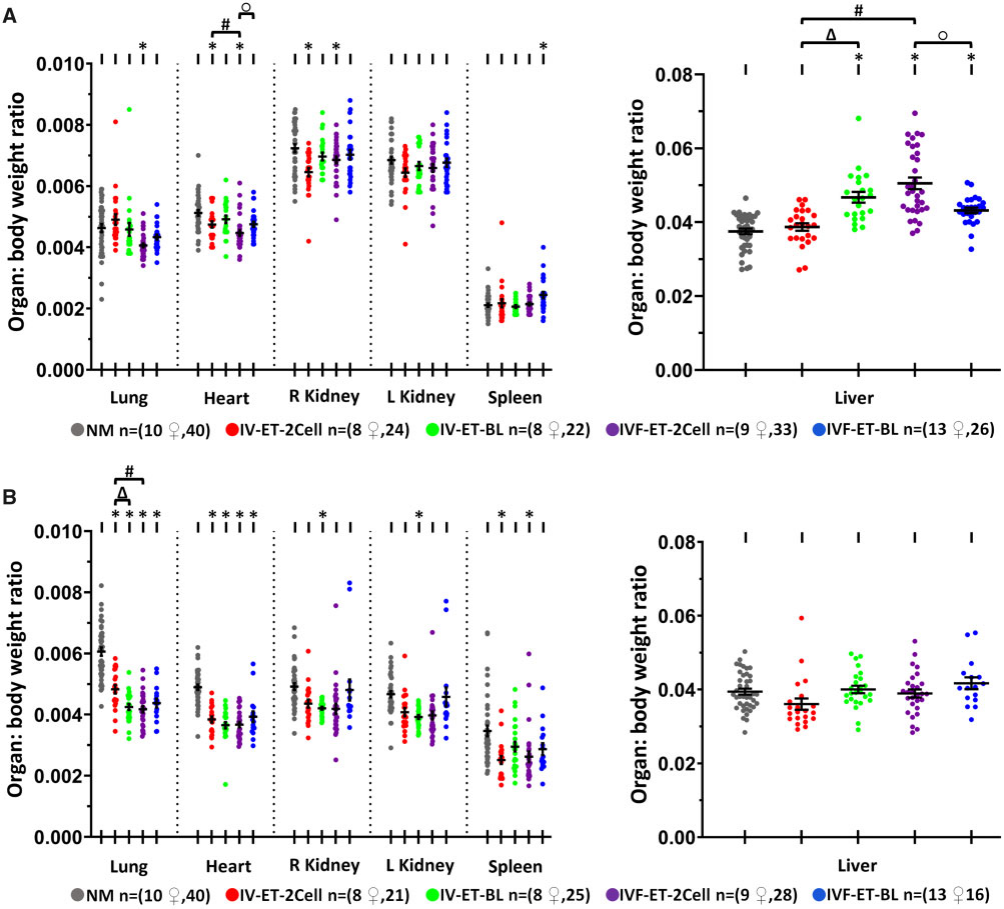
**Effect of ART techniques on organ/body weight in adult offspring.** Organ allometry variables in male (**A**) and female (**B**); mean (±SEM) organ: body weight ratio from 8 to 13 litters per treatment. Multilevel random effects regression analysis. *Indicates a significant difference (*P* < 0.05) between NM and selected groups, Δ indicates IV-ET-2-Cell vs. IV-ET-BL, # indicates IV-ET-2Cell vs. IVF-ET-2Cell, and ○ indicates IVF-ET-BL vs. IVF-ET-2Cell differences (*P* < 0.05). n mothers or foster mothers ♀, n offspring.

Offspring were sacrificed at postnatal Week 27 and organ/body weight ratios were determined before organ freeze storage. Male offspring organ weight was generally proportional to body weight but with exceptions (see Fig. 8 for details). Notably, IVF-ET-2Cell males had relatively smaller lungs, hearts and right kidneys and larger livers compared with NM males, whilst IVF-ET-BL males also had larger livers and spleens compared with NM males (Fig. 8A). IVF-ET-BL males had larger hearts and smaller livers than IVF-ET-2Cell males. The IV-ET-2Cell and IV-ET-BL control groups had few organ size differences from NM males. In contrast, female offspring from ART treatments generally had smaller proportioned organ sizes, especially lungs and hearts, compared with NM females but differences between the two IVF groups were not apparent (Fig. 8B). The combined techniques of ART (superovulation, IVF, culture, transfer) therefore contribute to altered organ allometry in both male and female offspring compared with natural unstimulated reproduction.

## Discussion

We have used an animal model to address the safety for long-term offspring health of specific ART techniques in common practice in clinics and in the absence of confounding parental infertility. Given the past record of adverse offspring health risk mediated through embryo culture (Cagnone and Sirard, 2016; Sunde *et al.*, 2016; Fleming *et al.*, 2018), the model was designed to distinguish specifically between short and long culture duration either up to cleavage-stage (two-cell) or blastocyst transfer, respectively. Both groups (IVF-ET-2Cell; IVF-ET-BL) were supported by direct *in-vivo* controls for transfer at these two stages (IV-ET-2Cell; IV-ET-BL) which included the background ART techniques (superovulation; ET) but in the absence of the tested techniques (IVF; short or long culture). These four groups were also compared with a NM group where no ART techniques were applied. Thus, the model is suitable for direct comparison of the health consequences for offspring arising from IVF and culture duration independent of other techniques, but also permits evaluation of the background techniques and the collective of all ART techniques. However, our design required the use of atmospheric oxygen rather than 5% for culture, although the former is reported to still be practised in some 40% of IVF cycles worldwide (van Montfoort *et al.*, 2020). This choice was necessary to maintain consistency between the two IVF groups and their two IV controls where embryo incubation was kept to an absolute minimum, essentially the time to complete ET in the surgery room, and could not be accomplished at 5% O_2_ for practicalities. Finally, the statistical approach of random effects regression analysis on the dataset permits outcomes to be evaluated in the entire offspring generated by each treatment rather than just on litter means, thereby integrating variability both within- and between-mothers and independent of the effect of litter size (Kwong *et al.*, 2004), as used in our previous periconceptional DOHaD models (Watkins *et al.*, 2008; Velazquez *et al.*, 2016, 2018).

One enduring feature of the dataset was the distinction between offspring phenotype arising from all four manipulated groups with that of the NM group. Thus, compared with the NM group, offspring from manipulated groups exhibited increased postnatal growth and poorer CV and metabolic health across the spectrum of assays undertaken, commonly in both male and female offspring at significant levels. This broad and unequivocal phenotypic consequence at one level demonstrates the collective effect of the ART techniques applied over the lifespan but is likely to be exaggerated because of the use of MF1 recipients for gestation and lactation. For example, it is established that the maternal uterine genotype of mouse recipients can influence offspring phenotype such as postnatal growth rate (Cowley *et al.*, 1989). Whilst we used inbred C57BL6/CBA embryos for genomic stability and capacity to overcome the ‘2-cell block’ in culture, outbred MF1 recipients were necessary to enhance pregnancy efficiency, a combination we have used successfully previously for DOHaD-related mouse studies (Velazquez *et al.*, 2016). Thus, the growth rate of offspring from the manipulated groups here broadly matched that previously reported (Velazquez *et al.*, 2016) and is similar to MF1 offspring from natural pregnancies (Watkins *et al.*, 2008) or slightly below that following MF1 embryo manipulations and transfer to MF1 recipients (Velazquez *et al.*, 2018).

In the critical group comparison of culture duration after IVF with all other ART techniques normalised, we found a curious dichotomy between IVF-ET-2Cell and IVF-ET-BL offspring, and particular males, in that CV outcomes (SBP; ACE activity; larger heart/body mass) were poorer in IVF-ET-BL treatments but conversely, metabolic outcomes (glucose response; raised basal insulin; increased liver/body mass; increased liver lipid accumulation) were poorer in the IVF-ET-2Cell group. Both CV phenotype in IVF-ET-BL and metabolic phenotype in IVF-ET-2Cell males were poorer than their respective controls (IV-ET-BL; IV-ET-2Cell) indicating outcomes were predominantly derived from IVF and culture duration, perhaps in combination with the timing of ET (discussed later), rather than by *in-vitro* manipulations and ET *per se*. To assess the basis for this dichotomy in health outcomes in IVF-ET-BL and IVF-ET-2Cell offspring, we first need to consider the direct effects of *in-vitro* culture on the early embryo.

Our study showed that *in-vitro* culture, although permissive for blastocyst formation, was suboptimal, slowing development and reducing proliferation of TE and ICM cells, as previously reported in other mouse ART models (Watkins *et al.*, 2007; Chen *et al.*, 2019). Culture conditions can interfere with two critical aspects of preimplantation development, namely embryo metabolism and the epigenetic regulation of the new embryonic genome. Embryo metabolism matures progressively from a low rate during fertilisation and early cleavage, dependent upon mitochondrial oxidative phosphorylation for energy production, and increases substantially at the blastocyst stage (Houghton *et al.*, 1996; Leese, 2012). This progression is accompanied by upregulated glycolysis in late cleavage stages, further enhancing energy availability for blastocyst morphogenesis, especially epithelial transport activity and increased protein synthesis for growth (Houghton *et al.*, 1996; Leese, 2012). Mitochondrial morphology also matures during cleavage with normal transverse cristae formation coinciding with the increased efficiency of ATP production at morula and blastocyst stages (Harvey, 2019). The unnatural metabolite milieu experienced in embryo culture can induce oxidative stress through increased production of reactive oxygen species alongside ATP in the mitochondrial electron transport chain (Takahashi, 2012; Cagnone and Sirard, 2016). Whilst natural protective mechanisms exist through antioxidant enzymes to maintain the redox balance, culture conditions can perturb this balance leading to impaired development affecting growth, gene expression and survival (Leese, 2012; Takahashi, 2012; Cagnone and Sirard, 2016). Indeed, direct manipulation of energy substrates, mitochondrial activity and redox potential in mouse zygotes leads to altered postnatal growth rates (Banrezes *et al.*, 2011). Furthermore, a range of environmental factors including maternal over-nutrition and obesity have also been shown to disturb mitochondrial functioning, localisation and mtDNA copy number in oocytes and early cleavage embryos with enduring effects on foetal and postnatal growth and metabolism (Igosheva *et al.*, 2010; Grindler and Moley, 2013; Wu *et al.*, 2015).

The second consequence of adverse culture environment is to interfere with the epigenetic reprogramming of the new embryonic genome (Chason *et al.*, 2011; Cagnone and Sirard, 2016; Sunde *et al.*, 2016). Global demethylation of the genome during cleavage is followed by a gradual, lineage-specific pattern of *de-novo* methylation initiated in the blastocyst to coordinate development (Seisenberger *et al.*, 2013). Thus, culture environment may alter the expression and methylation level of imprinted genes within the embryo persisting into later developmental stages (Doherty *et al.*, 2000; de Waal *et al.*, 2014). Non-imprinted genes are also vulnerable to culture conditions with the global pattern of gene expression (Feuer *et al.*, 2017) and DNA methylation distinct from that of *in-vivo* embryos (Wright *et al.*, 2011; Salilew-Wondim *et al.*, 2015; Canovas *et al.*, 2017). Epigenetic disturbance may at least partially derive from mitochondrial dysfunction since mitochondria supply intermediates in DNA methylation and histone acetylation through the 1-carbon metabolism pathway (Xu and Sinclair, 2015; Cagnone and Sirard, 2016; Ducker and Rabinowitz, 2017).

The poorer CV outcomes identified in IVF-ET-BL males after long culture versus both control IV-ET-BL and short culture IVF-ET-2Cell groups likely reflects the progressive negative effects of *in-vitro* culture on embryo metabolism and epigenetic stability. Indeed, we show a progressive increase in SBP in male offspring based upon the duration of culture from IV controls through to IVF-ET-BL offspring. Cardiac and associated vasculature form very early during development, from E8.5 in mouse, and is a complex morphogenetic process essential for embryo survival with recent research identifying significant epigenetic regulation (Kathiriya *et al.*, 2015). Adult CV dysfunction occurs in response to a wide range of peri-conceptional environments, indicating its sensitivity (Fleming *et al.*, 2018). Moreover, an epigenetic basis for adverse CV health including arterial hypertension has been reported in a mouse ART model, mediated through altered DNA methylation of the endothelial eNOS gene in the aorta, leading to reduced eNOS expression and disturbed NO signalling (Rexhaj *et al.*, 2013). Notably, the CV phenotype and associated epigenetic alteration in the eNOS gene can be prevented by inclusion of the epigenetic regulator, melatonin, in embryo culture medium (Rexhaj *et al.*, 2015). Indeed, the significant positive correlation identified between SBP and lung ACE level in the IVF-ET-BL males, but not other groups, suggests that ACE expression, known to be epigenetically regulated (Mudersbach *et al.*, 2019) and sensitive to the peri-conceptional environment (Watkins *et al.*, 2006, 2007), may contribute an epigenetic pathway to affect later CV health.

In contrast to the clear link between extended culture and offspring CV dysfunction, it was the IVF-ET-2Cell group with shorter culture duration that lead to the poorer metabolic phenotype in offspring. Collectively, male offspring from this treatment demonstrated poorer glucose handling which correlated negatively with body mass, increased basal insulin levels, increased relative liver sizing and liver lipid accumulation, compared with either the direct control group (IV-ET-2Cell) or the IVF-ET-BL group. Increased birth weight and poorer glucose and insulin regulation were previously reported in mouse IVF offspring following ET at the two-cell stage but predominantly in females (Scott *et al.*, 2010). Similar poorer glucose handling mainly in female offspring following IVF was found after mouse blastocyst ET and coincided with metabolic dysfunction across several tissues including liver, evidenced by microarray analysis (Feuer *et al.*, 2014). Furthermore, liver metabolic dysfunction including accumulation of monounsaturated fatty acids has been reported following mouse IVF and ET at the two-cell stage (Wang *et al.*, 2013). Mouse IVF also leads to increased phospholipid accumulation in foetal liver (Li *et al.*, 2016), indicating prenatal origin of ART-mediated metabolic impairment. Given the increased accumulation of lipid in the male IVF-ET-2Cell liver, it would be interesting in future studies to determine serum lipid levels and adipose tissue composition for a broader understanding of lipid dysregulation in this group. The co-occurrence of markers of metabolic disease risk in several studies, as well as in our current study, confirm the link between ART and adult metabolic health.

This distinction in outcomes between IVF-ET-2Cell and IVF-ET-BL groups suggests different mechanisms and biological pathways may be at work for metabolic and CV outcomes. Apart from the shorter culture duration, the IVF-ET-2Cell group experienced ET during the two-cell stage when the mouse embryonic genome is predominantly activated (EGA) (Flach *et al.*, 1982). The period of EGA at the transition from maternal to embryonic control of development is recognised as one of particular sensitivity to culture conditions across mammalian species, affecting embryo potential (Lonergan *et al.*, 2003; Zander *et al.*, 2006) and is discussed in detail elsewhere (Brison *et al.*, 2014). A convincing argument suggests that stressful manipulations during the EGA (such as ET here) may be accentuated by the absence of gap junction communication between blastomeres to coordinate homogeneity and protection in intercellular maturation (Brison *et al.*, 2014). EGA in the human occurs slightly later in cleavage, at the 4- to 8-cell transition (Braude *et al.*, 1988; Vassena *et al.*, 2011), but cleavage ET in human ART normally coincides with this cellular stage.

A further characteristic of our study has been the clear disparity in outcomes based upon offspring sex with males being far more sensitive that females. Sexual dimorphism has been commonly found in periconceptional DOHaD programming studies in response to diverse challenges including ART-based models and evident in small and large mammals and humans (Hansen *et al.*, 2016; Fleming *et al.*, 2018). In mouse studies of embryo culture effects on offspring cardiometabolic health, males commonly show increased sensitivity, as here (Donjacour *et al.*, 2014; Velazquez *et al.*, 2018), but female vulnerability has been shown elsewhere (Feuer *et al.*, 2014), indicating that strain differences may be contributory. This also likely reflects different susceptibilities to CV disease based on sex, which arise *in utero* (Schalekamp-Timmermans *et al.*, 2016). Environmental conditions such as nutrient and metabolite levels both *in vivo* and *in vitro* can differentially influence embryo response in terms of signalling activity, gene expression and morphogenesis in a sex-specific manner that can persist through gestation and postnatal life (Hansen *et al.*, 2016).

## Conclusion

We have shown that IVF and embryo culture in a mouse model specifically associate with adverse CV and metabolic outcomes particularly in male offspring independent of background superovulation and ET techniques. Our study shows a clear effect of culture duration after IVF with long culture to the blastocyst stage before ET leading to a poorer CV phenotype and shorter culture to the two-cell stage before transfer resulting in a poorer metabolic health phenotype. We consider this distinction in outcome likely reflects different pathways leading to these health conditions initiated from preimplantation environment and the interaction between culture duration and the timing of ET in relation to EGA. These findings further pinpoint the risks of preimplantation manipulations in the programming of long-term health outcomes. From a clinical perspective, whilst our data do not identify a safer strategy for IVF and culture duration, they do show the biological and health implications that derive from the ART culture protocol.
